# A profile and approach to chronic disease in Abu Dhabi

**DOI:** 10.1186/1744-8603-8-18

**Published:** 2012-06-27

**Authors:** Cother Hajat, Oliver Harrison, Zainab Shather

**Affiliations:** 1 Department of Public Health & Research, Health Authority Abu Dhabi, Airport Rd, PO Box 5674, Abu Dhabi, United Arab Emirates

## Abstract

As a country, the United Arab Emirates has developed very rapidly from a developing country with a largely nomadic population, to a modern and wealthy country with a Western lifestyle. This economic progress has brought undoubted social benefits and opportunities for UAE citizens, including a high and increasing life expectancy. However, rapid modernization and urbanization have contributed to a significant problem with chronic diseases, particularly obesity-related cardiovascular risk. In response the Health Authority of Abu Dhabi has significantly strengthened its data systems to better assess the baseline and measure the impact of targeted interventions. The unique population-level Weqaya Programme for UAE Nationals living in Abu Dhabi has recruited more than 94% of adults into a screening programme for the rapid identification of those at risk and the deployment of targeted interventions to control that risk. This article describes the burden of non-communicable disease in Abu Dhabi, and the efforts made by the Health Authority of Abu Dhabi to tackle this burden including the development of a whole population cardiovascular screening programme changes to health policy, particularly in terms of lifestyle and behaviour change, and empowerment of the community to enable individuals to make healthier choices. In addition, recommendations have been made for global responsibility for tackling chronic disease.

## Introduction

The growing problem of chronic disease is well documented. The World Health Organisation (WHO) stated in 2008 that “Today, non-communicable diseases (NCDs), mainly cardiovascular diseases, cancers, chronic respiratory diseases and diabetes represent a major threat to human health and development. These four diseases are the world’s biggest killers, causing an estimated 35 million deaths each year - 60% of all deaths globally - with 80% in low- and middle-income countries” [1].

Data consistently show a rapidly rising global burden, placing chronic disease near the top of the global risk landscape in terms of both likelihood and severity [2]. Data from Abu Dhabi, the largest of the seven emirates making up the United Arab Emirates (UAE) show diseases of the circulatory system consistently make the greatest contribution to emirate mortality, accounting for at least 27% of deaths in 2010 despite its young population (median age 18 for Nationals, 31 for Expatriates) [3] and with the second highest prevalence rate for diabetes in the world (Diabetes Atlas, International Diabetes Federation) [4].

The key challenge for chronic disease is the development of effective and scalable interventions. In line with recommendations for global action by the WHO [5], World Economic Forum [6] and Institute of Medicine [7] reports, this chapter sets-out a whole population cardiovascular screening programme, the Health Authority of Abu Dhabi set out its response to health policy, particularly in terms of lifestyle and behaviour change. One such intervention was the Weqaya programme, a population level cardiovascular disease intervention. In addition, this paper also outlines recommendations for global responsibility for tackling chronic disease.

## Background

The United Arab Emirates (UAE) is a small, relatively young country, having established independence 40 years ago, in 1971. The pace of change in the past four decades - including the striking rate of growth and urbanization - has been tremendous, transforming the UAE into a modern, wealthy, and prosperous country.

Abu Dhabi is the capital of the United Arab Emirates (UAE). It is the largest of the seven emirates that comprise the UAE, representing approximately 87% of the UAE’s total land area [8]. The Emirate is made up of three regions; Abu Dhabi city, Al Ain (Eastern region) and the Western region.

Prior to the discovery of oil, the U.A.E. economy was reliant on pearl trading, herding, fishing and agriculture. During the oil boom in the 1970’s, the petroleum industry started to dominate the economy with Abu Dhabi becoming one of the largest exporters of oil globally. Abu Dhabi holds 9% of the world’s proven oil reserves, and 5% of the world’s natural gas reserves [8]. The Emirate produces an estimated 2.5 million barrels of oil per day, accounting for 90% of the UAE’s oil production [8]. However, Abu Dhabi is actively attempting to diversify its economy through investments in the tourism, infrastructure, financial and real estate sectors. In 2010, the non-oil sector contributed 50.3% to Abu Dhabi’s gross domestic product of AED 622, 316 million (USD 169,453 million) [8]. Abu Dhabi has a gross domestic product per capita of AED 315,300 (USD 85,854 million), one of the highest in the world [8].

Following the discovery and export of oil, Abu Dhabi’s population grew rapidly. It is the second most populous city after Dubai, representing approximately 33% of the UAE’s total population [8]. Abu Dhabi has a population of 2.3 million of whom 20% are native Emirati, and an overwhelming 80% are a mix of Western, Arab and Asian Expatriates [3]. Abu Dhabi has a relatively young working population with 66% of Emiratis under the age of 30 compounded by the fact that expatriate residency is dependent on employment [3]. Of the 2.3 million residents, the majority (65%) live in urban areas [9].

Although this rapid urbanization and economic growth has clearly provided social benefits and opportunities for UAE citizens, it has also led to largely unhealthy lifestyles characterized by lack of physical activity, tobacco consumption and poor diets. These modifiable NCD risk factors have significantly contributed to the large chronic disease burden which faces the relatively young Abu Dhabi population.

### The non-communicable disease burden in Abu Dhabi

A wide-ranging benchmarking review (the Global Health Index) produced by management consultants (McKinsey and Company) in 2006 reported on health infrastructure, interventions, risk factors and outcomes, relative to other countries largely using data from the WHO (2000–2002 estimates) [10]. It suggested significant issues at the UAE national level particularly with respect to chronic disease as shown in Table 1, with high burden from cancer, cardiovascular and diabetes, high mean BMI and high rates of smoking among males.

**Table 1:** **Overview of UAE health status **[7]** DALY: disability adjusted life years**

***Domain***	***Indicator***	***Abu Dhabi value***
***Infrastructure***	No. physicians per 1000 habitants	2
	% out of pocket expense	18
***Interventions***	% population with access to clean water	100
	MCV vaccination rate %	94
	DTP vaccination rate %	94
	BCG vaccination rate %	98
	Change in HIV prevalence over last 2 years	Not applicable
***Disease specific outcomes***	Total infections DALY	226
	***Total cancer, cardiovascular and diabetes DALY***	1594
***Risk factors***	Mean BMI (adults)	29 Kg/m^2^
	Smoking prevalence	9% (24% males, 1% females)
	Excess alcohol consumption	2.8%

**Figure 1:**
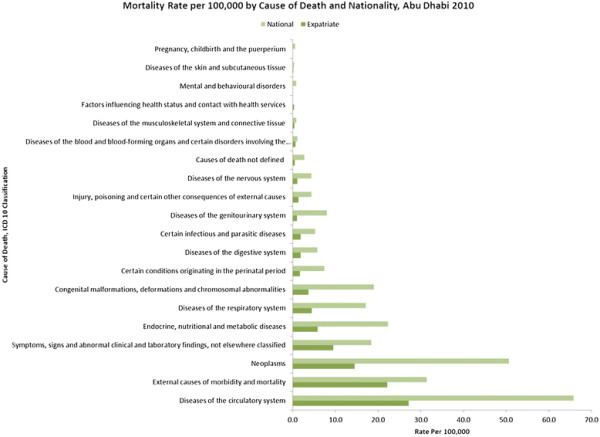
**Cause-specific death rates for Abu Dhabi 2010**[3]**.**

The 2010 Abu Dhabi mortality data [3] (Figure 1) shows that the top cause of mortality for both Nationals and Expatriates are diseases of the circulatory system.

**Figure 2:**
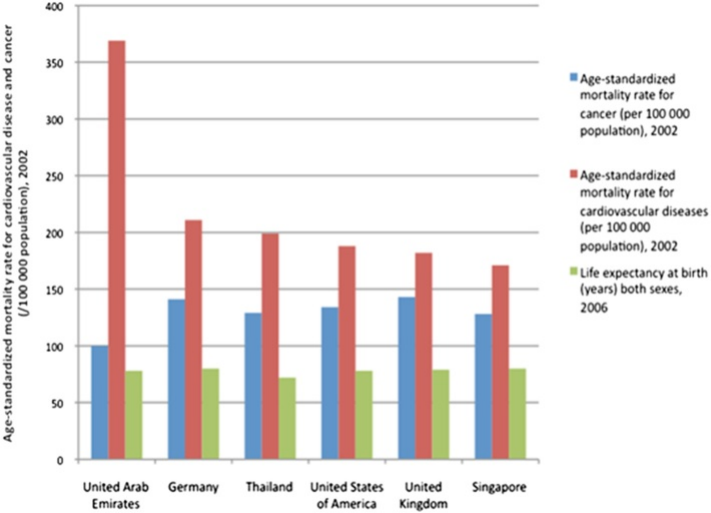
**A comparison of UAE age-standardised mortality rates for cardiovascular disease and cancer and life expectancy at birth WHOSIS data**[12]**.**

The young working age expatriate population is not the target age for the most common causes of mortality such as cardiovascular events and cancers. Congenital abnormalities are much higher in Nationals (19/100,000 population) than in Expatriates (4/100,000 population) which has been attributed to the high rates of consanguinity in the UAE [11]. Figure 2 shows that the age-standardised mortality rates for cardiovascular disease are significantly higher in the UAE compared with other benchmark countries, whereas for cancer they are lower.

Hence, whilst cancer is within the top ten priorities for Abu Dhabi as it falls within the top 3 causes of mortality for Nationals, cardiovascular disease warrants the highest priority due to its high burden of mortality and discrepancy in age-standarised mortality rate between UAE and elsewhere.

**Figure 3:**
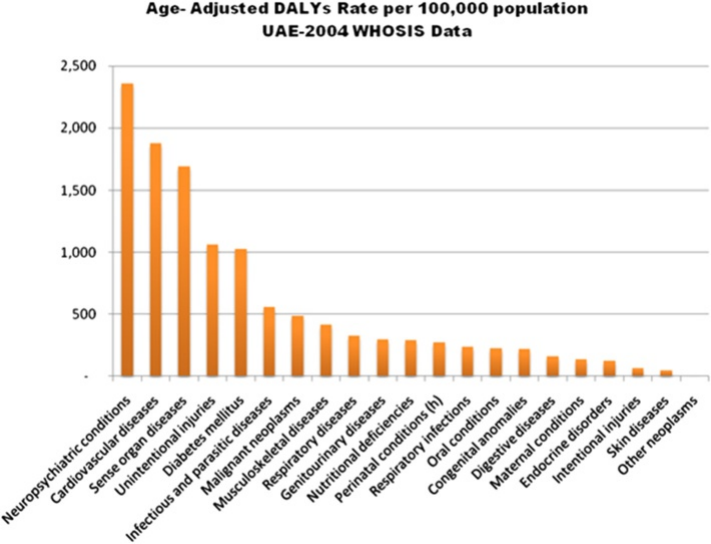
**Age-adjusted disability adjusted life years (DALYs) expressed as a rate per 100,000 population by disease area, UAE 2004 WHOSIS data**[12]**.**

If morbidity is also included in the burden, other chronic disease areas warranting intervention also become apparent such as neuro-psychiatric disorders, as can be seen in Figure 3.

The mortality, morbidity, together with the feasibility of achieving impact are all used to develop the Public Health priorities for the Health Authority Abu Dhabi, which are in order of priority:

1. CVD prevention & management

2. Road Safety

3. Tobacco Control

4. Cancer Control

5. Mental Health

6. Mother, Infant and school health

7. Musculo-skeletal health

8. Occupational & Environmental Health

9. Infectious Disease

10. Oral Health

Chronic diseases are represented in 6 out of the 10 priority areas and are clearly an important area to tackle for dealing with the increasing demand on healthcare burden from chronic disease.

### Specific data on cardiovascular disease

#### Adults

In April 2008, a National cardiovascular screening programme, Weqaya, meaning ‘protection’ in the Arabic language, commenced, screening over 94% of the population of Abu Dhabi adult National residents to date [13]. Currently the Weqaya screening programme screens only Nationals and not non-National residents in the UAE.

**Figure 4:**
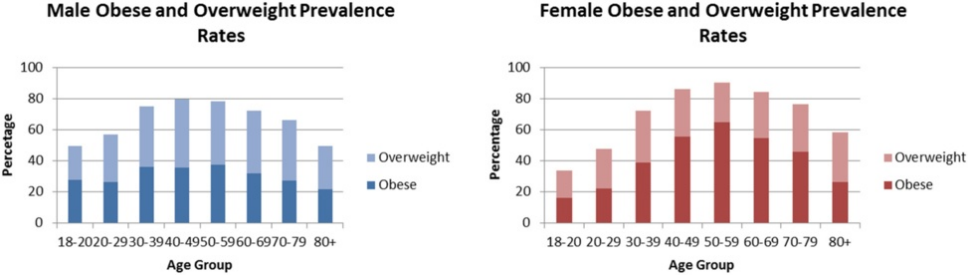
**A comparison of rates of obesity and overweight by age group and gender**[14]**.**

**Figure 5:**
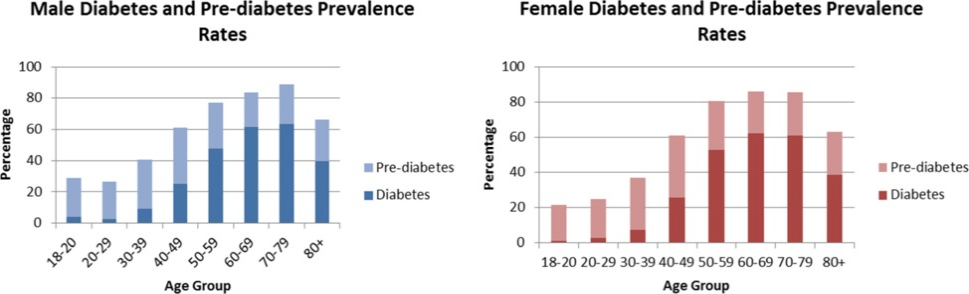
**A comparison of rates of diabetes and pre-diabetes by age group and gender**[14]**.**

Of 173,501 screened subjects, 50,138 were included in an initial analysis with mean age (SD) of 36.8 (14.3) years [13]. Rates of several of the cardiovascular risk factors were extremely high. Figures 4 and 5 show the distribution of obesity and diabetes by age and gender; 67% of adults were either overweight or obese (using BMI) and 57% had central obesity (using Waist Hip Ratio (WHR)) [13]. Diabetes was present in 18% of the population and a further 27% had evidence of pre-diabetes [13]. Yet rates of some risk factors such as hypertension and smoking were found to be lower than rates seen in Western populations at 23% and 12%, respectively.

An unpublished household survey in Abu Dhabi in 2005 including both Nationals and non-Nationals also found excessively high rates of most risk factors for cardiovascular disease [3]. This was a household and opportunistic survey and so is likely to be less representative of the population, with a combination of self-reported and measured risk factors. It found similarly high rates of obesity in the nationals as for the Weqaya programme, with obesity rates at 36% in Nationals and 20% in non-Nationals. Diabetes and dyslipidaemia were also higher in Nationals at 21% and 36% than in non-Nationals at 18% and 18%. Hypertension and smoking rates were lower in Nationals at 17% and 11% compared with non-Nationals at 35% and 25%. The results in the Nationals were very similar to those found in the Weqaya programme. Perhaps more surprising are the excessive rates of certain risk factors in the non-Nationals. This is likely to be a result of the predominance of South East Asian Expats, many of whom originate from countries with high rates of cardiovascular risk such as diabetes and smoking. The non-National population are also older than the Nationals (median age of 31 compared with 18 years, respectively) [3] which would partly account for the higher rate of hypertension.

#### Children

Rates of obesity are increasing in children in the UAE; children who are obese are far more likely to have abnormal weights as adults. This is a major risk factor for cardiovascular disease. The WHO Global Schools Health Survey in children aged 13–15 years of all nationalities reported that in the UAE, obesity rates (≥95^th^ Centile) were 12% in 2005 and 14% in 2010 [15]. Overweight (≥85^th^ Centile) was present in 33% in 2005, [14] increasing to 43% by 2010. [15] . In order to monitor and better manage the increasing rate of abnormal weight in children, since January 2010 all school children in Abu Dhabi have been required to undertake annual BMI screening under Health Authority Abu Dhabi (HAAD), with specialist referral and further investigation and treatment for those at high risk (≥85^th^ Centile). Clinical care pathways and clear standards of care are stipulated and mandated for weight management in children and adults and are made available online as well as through CME accredited training for healthcare providers in Abu Dhabi.

#### The drivers

To date there has been somewhat limited analysis of the drivers of chronic disease in the region, but these are likely to be due mostly to changes in the lived environment, driven by four factors. These are:

1. Developmental [16]:

· Very rapid processes of modernization and urbanization (driven by rapid economic development due to petrochemical wealth).

· Rapid increase in population size.

· Profound generational change in lifestyle from a nomadic population to a modern Western lifestyle.

2. Cultural:

· Traditional Khaleeji Arab clothing limits direct feedback of weight-gain and possibly of social feedback.

· Enduring stigma associated with exercising outdoors, especially for women.

· Historic lack of role models in sports.

· Historic lack of sports culture, with only a very recent widespread interest in participating and watching team sports.

3. Economic:There is to date no rigorous evidence of an inverse relationship between socioeconomic status and CVD risk, although migrant workers on lower salaries are often employed in manual labour. In turn, as a programme of interventions is formed, this means that a different approach is required to population level interventions such as pricing policy for the promotion of healthy choices.

4. Environmental drivers:

· The hot climate limits the availability of outside exercise for 3–4 months over the summer.

The drivers described above have contributed to an escalating prevalence of risk factors for chronic diseases. The examples of obesity and smoking as risk factors are elaborated on below.

### Example 1: Obesity

The drivers above have combined to create a significant prevalence of obesity.

**Figure 6:**
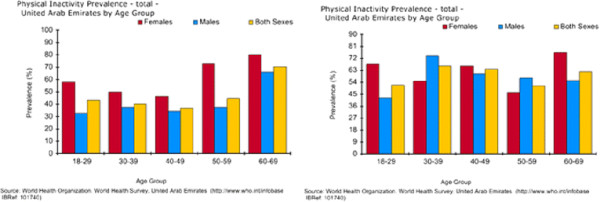
**Results of a WHO survey on prevalence of physical inactivity**[17]**, a. Urban area, n = 861, b. Rural area, n = 243.**

Figure 6 shows results from a UAE survey on levels of physical inactivity, comparing urban and rural residents [17]. It reported that in urban areas around 40% of adults aged under 30 years reported taking no exercise but this increased to around 70% of adults by the age of 60 years. Perhaps more striking is that physical inactivity was much higher in women at all ages with 60% of young women self-reporting that they take no exercise. As these are self-reported, actual rates of physical inactivity are likely to be higher.

### Example 2: Smoking

Smoking rates are very high in young National males with 16% of 18–20 year olds, 27% of 20–29 year olds and 28% of 30–39 year olds admitting smoking in the Weqaya screening programme and is likely to be an under-representation of non-cigarette tobacco use. Smoking rates in males are higher in the UAE than in some GCC countries such as Bahrain, Saudi Arabia and Oman but considerably lower than the majority of other Middle Eastern countries where rates are mostly in excess of 30%. Smoking in females is lower in the UAE than any other Middle Eastern country [18]. The overall smoking rate in the UAE of 11% in the Weqaya population is considerably lower than 2007 rates in the UK at 22% [19] and the US of 20% [20].

However rates of non-cigarette tobacco use are likely to count for a high proportion of tobacco consumption in the UAE as well as many other Middle East countries. Whilst years of education and awareness campaigns have succeeded in spreading the message on negative health effects of cigarette smoking, the same is not true for the relatively new phenomenon of water-pipe (shisha) use. The negative effects of shisha have been demonstrated such as on tar and nicotine intake [21], risk of lung cancer, respiratory illness and low birth-weight [22], but on the whole studies remain sparse and the body of evidence is inadequate to form concrete conclusions about the level of risk.

### Social, health systems and economic impacts

#### Mortality and morbidity

**Figure 7:**
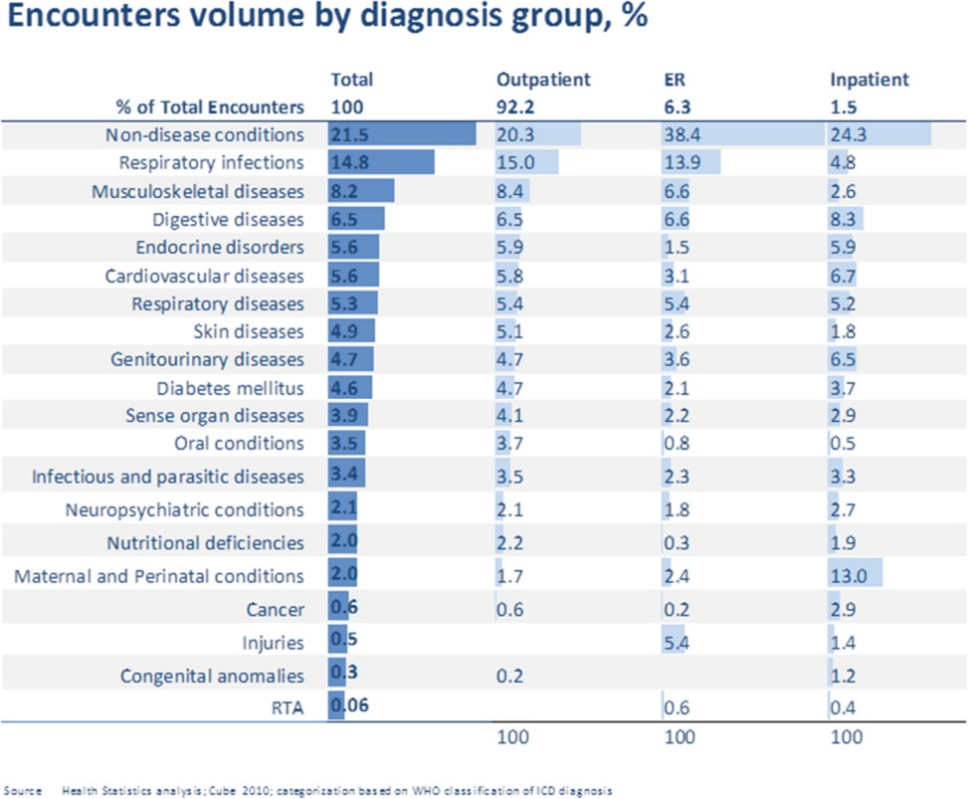
**Encounters in Abu Dhabi, 2010 by diagnosis and type of healthcare facility Source: HAAD statistics**[3]**.**

Mortality and morbidity have been described in Table 1 and Figures 1,2,3,4,5,6 and 7 above. The population of Abu Dhabi is relatively young with increasing life expectancy (reported to be 73 by WHO in 2005 and 78 in 2009) [12]. Despite this, chronic disease, particularly diseases of the circulatory system, consistently make the greatest contribution to mortality. Therefore, the impact on both actual numbers of life years lost and quality of life due to the chronic diseases are likely to be high.

#### Healthcare facility encounters

Figure 7 provides a snapshot of morbidity for the population of Abu Dhabi by reason for encounters by diagnosis group and type of facility. It shows that chronic conditions other than cardiovascular disease also put a significant burden on the healthcare system. Respiratory infections were the most common diagnosis group overall by far, followed by musculo-skeletal disease and Digestive diseases. Endocrine diseases encounters were similar in volume to cardiovascular disease.

#### Financial

Chronic disease and in particular, cardiovascular disease, costs far outweigh costs of other disease groups and the costs of their prevention and screening programmes. Costs of diabetes in Abu Dhabi have been estimated in a recent small study in Al Ain, Abu Dhabi [23],. The study enrolled a sample of 150 patients with diabetes mellitus in 2004–2005, and measured their medical costs over the ensuing 12 months. The costs were then extrapolated to the population of the UAE using conventional and inference statistics. The average annual cost of diabetes care was estimated to be $3,995 per patient, rising to as high as $6,175 in patients who have developed complications. Combining the Weqaya prevalence rate of 18% of the UAE population with diabetes with the mean cost of $3,995, diabetes may be currently costing up to $1.1 billion per year in Abu Dhabi alone, with this being set to rise significantly. Added to this are the indirect costs of loss of productivity, impact on carers and early mortality.

Like many reports preceding it, the World Economic Forum in 2008 highlighted the business case for governments to do more to prevent chronic disease [24]. With globalization the increase in chronic disease has been even greater in emerging economies with countries such as Brazil, China, Russia and India currently losing more than 20 million productive life-years annually to chronic disease; that number is expected to increase by at least 65% by 2030. With this serious threat to global financial stability, governments have a clear imperative to prevent chronic disease for four major reasons as highlighted in the World Economic Forum report:

1. Chronic Disease Drives Healthcare Costs

· In the US alone, people with chronic disease account for more than 75% of the nation’s US$ 2 trillion in medical spending.

· The most costly conditions and health risk factors related to productivity are different from those when considering only the cost of treating the disease. For example, depression has amongst the largest impact on productivity and therefore loss in productivity.

2. Productivity Losses Associated with Chronic Disease Are Even Greater

· Productivity losses associated with workers with chronic disease are as much as 400% more than the cost of treating chronic disease.

· Table 2 shows the costs due to loss of productivity by GDP comparing various countries.

3. Workplace Wellness Efforts Can Positively Impact Human Capital Investments

· By helping employees to have more productive lives, organizations can protect this asset in the face of growing labour shortages globally.

· An organization that shows that it values its workers is more likely to attract, retain and motivate employees. Leading organizations have utilized prevention and wellness programmes to demonstrate the value they place on their workers.

4. Sustainability Is Threatened by the Epidemic of Chronic Disease

· The epidemic of chronic disease - a product of both environment and behaviours - is a social phenomenon that is as equally prevalent and preventable as issues such as global warming, infectious diseases, poverty, terrorism, unsanitary water and basic infrastructure, many of which are also inter-linked with chronic diseases.

**Figure 8:**
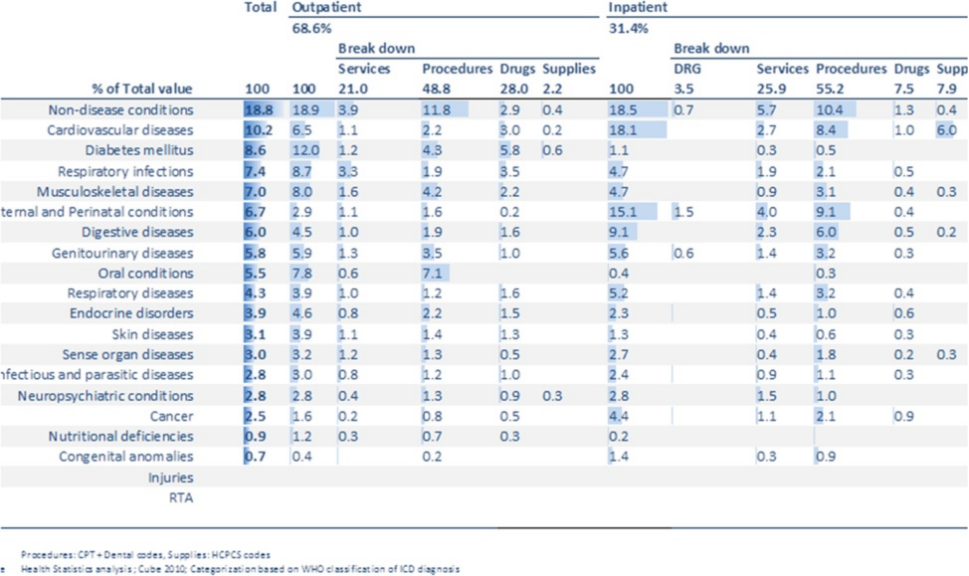
**Encounters in Abu Dhabi, 2010 by value Source: HAAD statistics**[3]**.**

Figure 8 gives a snapshot of morbidity for the population of Abu Dhabi by reason for encounters by value. It shows that cardiovascular diseases and diabetes/endocrine diseases together account for at least 18.8% of all encounters by cost.

**Table 2:** **Projected loss of income (expressed as %GDP) in 2005 and 2015 in selected countries attributable to chronic disease **[25]

	**Year**	**Brazil**	**Canada**	**China**	**India**	**Nigeria**	**Pakistan**	**Russia**	**United Kingdom**	**United Republic of Tanzania**
	*Estimated loss in GDP/Projected GDP (%)*									
**NCD deaths**	**2005**	0.19%	0.07%	0.31%	0.35%	0.23%	0.30%	1.13%	0.10%	0.29%
	2015	0.48%	0.15%	1.18%	1.27%	0.65%	1.02%	5.34%	0.32%	0.86%
*Mean*		*0.3%*	*0.1%*	*0.7%*	*0.7%*	*0.4%*	*0.6%*	*2.7%*	*0.2%*	*0.5%*
All-cause deaths	**2005**	0.92%	0.38%	1.08%	1.27%	1.11%	0.84%	2.73%	1.11%	1.57%
	2015	3.21%	0.64%	3.94%	5.04%	3.07%	3.08%	12.35%	5.18%	4.19%
*Mean*		*1.4%*	*0.4%*	*1.6%*	*2.0%*	*1.6%*	*1.3%*	*4.5%*	*1.8%*	*2.2%*

## Chronic disease control efforts in Abu Dhabi

A dedicated Public Health department was created in Abu Dhabi in 2006 to understand and tackle the burden of disease in the Emirate. The department began working rapidly to improve the quality of healthcare data within the Emirate, particularly in the reporting of vital statistics in line with international standards such as WHO guidelines [26]. Alongside the public health datasets, the Health Authority has created a data system (the Knowledge Engine for Health, “KEH”) which uses e-claims, under mandatory health insurance, to collect systematic health facility episode data, creating from mid2008 strong prospective data for the entire population of the emirate, automated within the healthcare infrastructure to ensure its sustainability.

The Public Health department has worked to quickly understand the burden of disease in the population, and outline public health priorities which urgently needed to be addressed. Cardiovascular disease prevention was identified as the number one public health priority, and hence an Emirate-wide screening programme, Weqaya, was developed.

### Example of the Abu Dhabi approach to CVD prevention: The weqaya screening programme

A good example to take for Abu Dhabi is the Health Authority Abu Dhabi response to CVD prevention. WHO data from the year 2000 and smaller individual reports indicated a worrying cardiovascular burden of disease in the UAE. The response was to establish a national CVD screening programme, the Weqaya screening programme, that screened for all ‘Framingham’ CVD risk factors for Emirati adults in Abu Dhabi [13,27]. The justification and utility for this programme is in definitively establishing the epidemiological profile of the problem, establishing a cohort for follow-up and for establishing a chronic disease model for the primary prevention, secondary prevention and treatment of cardiovascular disease. The premise for this was the overwhelming evidence of clinical and cost-effectiveness of screening and early detection of cardiovascular risk factors.

**Figure 9:**
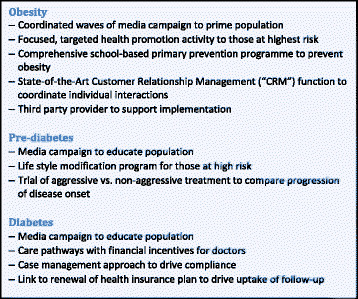
Summary elements of the HAAD CVD education and awareness campaign.

If we take Type II diabetes as an example, early detection in the best case scenario of diagnosing the condition 10 years earlier than clinical onset has been shown to reduce the risk of a cardiovascular event to 50% (clinical quality) and predicted to reduce the cost of healthcare in these individuals to one third (cost-effectiveness) [28]. The Weqaya programme was supported by a targeted, interactive and high-impact health promotion campaign that focused on empowering the public to take action as detailed in Figure 9.

### Society response: “health policy agenda”

The Abu Dhabi approach to health recognises that the most influential factor in changing behaviour at the level of the individual is to make the healthier choices easier and by default, make unhealthy choices harder. This necessitates a series of measures that employ a policy change as well as the empowerment of individuals and encouragement to make healthier choices. The involvement of multiple health and non-health, public and private sectors necessitates wide stakeholder engagement from the outset. HAAD has identified key elements to ensure that the HAAD ***health policy agenda*** is successful. The HAAD Health Policy Agenda aims to create a “healthy Abu Dhabi” by reducing harmful exposures and encouraging healthy behaviour through a number of initiatives, for example ensuring availability of active transport and leisure facilities to encourage physical activity, food labelling to encourage healthy eating and smoke free workplaces. In addition, HAAD is empowering individuals to look after their own health through, for example, providing education on healthy living and educating the community on the availability of gyms, football fields and other green areas. HAAD ensures that the health policy agenda is aligned across the Abu Dhabi Emirate by measuring standard metrics at individual and group level with central reporting to HAAD.

## Government regulation

Population level policy can bring about changes in lifestyle behavior, which even if small in degree, can deliver significant impact with relatively short timescales. However, population level policy change affecting lifestyle choice attracts significant push-back from sectors with dis-incentives for change. The food industry is a prime example where evidence based interventions such as reductions in salt, sugar and fat content of daily nutritional intake have been recommended for many years with failure to act in a timely manner. Denmark have recently become the first country to ‘tax’ foods high in fat content; The tax rate is 16 Danish kroner (approximately USD 3) per kilogram of saturated fat for all foods which contain more than 2.3% saturated fat [29,30]. The tobacco industry is another example where the deployment of clearly evidence-based public health interventions have failed to be implemented at global level despite the concept of a global WHO Framework Convention on Tobacco Control (WHO FCTC). The WHO Framework Convention on Tobacco Control is an international evidence-based treaty whose objective is to protect present and future generations from the devastating health, social, environmental and economic consequences of tobacco consumption and exposure to tobacco smoke by providing a framework for tobacco control measures to be implemented at the national, regional and international levels in order to continually and substantially reduce the prevalence of tobacco use and exposure to tobacco smoke [31].

## Recommendations

A multi-sectorial approach at the international and national levels is key to achieving impact for chronic disease as has been demonstrated in successful communicable disease programmes such as for HIV and malaria [32,33].

**Figure 10:**
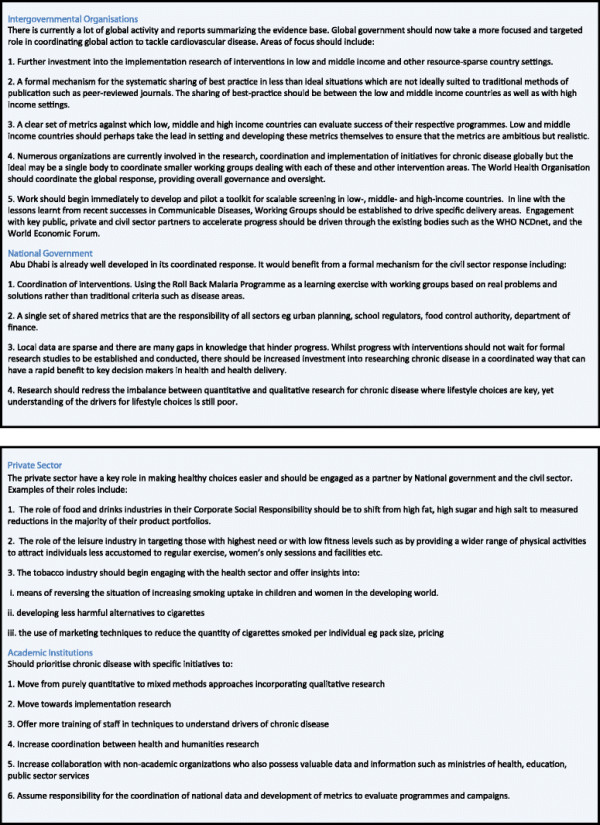
Recommendations for global responsibility in tackling chronic disease.

Figure 10 describes recommendations for responsible and relevant sectors in the fight against chronic disease.

## Conclusions

The United Arab Emirates has developed very rapidly from a developing country with a largely nomadic population, to a modern and wealthy country with a Western lifestyle. This economic progress has brought undoubted social benefits and opportunities for UAE citizens, including a high and increasing life expectancy. However, rapid modernization and urbanization have contributed to a significant problem with chronic diseases, particularly obesity-related cardiovascular risk.

In response the Health Authority of Abu Dhabi has significantly strengthened its data systems to better assess the baseline and measure the impact of targeted interventions. Prevention programmes are being developed, the impact of which can be measured directly through the healthcare data system.

Lifestyle and behavior changes are important in reducing the incidence of cardiovascular disease and its risk factors. The Abu Dhabi approach to health recognises that the most influential factor in changing behaviour at the level of the individual is to make the healthier choices easier and make unhealthy choices harder. This is being achieved through health policy which mandates improved nutrition, urban planning and environment as well as the empowerment of individuals and encouragement to make healthier choices. This requires the involvement of multiple health and non-health sectors from the outset.

However, in addition to efforts made at country level, in order to effectively tackle the burden of chronic disease, a multi-sectorial approach at both the international and national levels is critical.

## Competing interests

The authors declare that they have no competing interests.

## Authors’ contributions

CH designed the study, data analysis and interpretation and wrote the manuscript. OH contributed to writing of the manuscript. ZS contributed to the data analysis, interpretation and writing of the manuscript. All authors read and approved the final manuscript.
